# Evidence for Vpr-dependent HIV-1 Replication in Human CD4^+^ CEM.NKR T-Cells

**DOI:** 10.1186/1742-4690-9-93

**Published:** 2012-11-07

**Authors:** Tao Zhou, Ying Dang, Jacob J Baker, Jiajun Zhou, Yong-Hui Zheng

**Affiliations:** 1 Department of Microbiology and Molecular Genetics, Michigan State University, 567 Wilson Road, 2215, Biomedical and Physical Sciences Building, East Lansing, MI, 48824-4320, USA

**Keywords:** Vpr, Vpx, Host Restriction, HIV-1, SAMHD1, G2 cell cycle arrest, DCAF1

## Abstract

**Background:**

Vpr is exclusively expressed in primate lentiviruses and contributes to viral replication and disease progression in vivo. HIV-1 Vpr has two major activities in vitro: arrest of cell cycle in the G2 phase (G2 arrest), and enhancement of viral replication in macrophages. Previously, we reported a potent HIV-1 restriction in the human CD4^+^ CEM.NKR (NKR) T cells, where wild-type (WT) HIV-1 replication was inhibited by almost 1,000-fold. From the parental NKR cells, we isolated eight clones by limiting dilution. These clones showed three levels of resistance to the WT HIV-1 infection: non-permissive (NP), semi-permissive (SP), and permissive (P). Here, we compared the replication of WT, Vif-defective, Vpr-defective, and Vpu-defective viruses in these cells.

**Results:**

Although both WT and Vpu-defective viruses could replicate in the permissive and semi-permissive clones, the replication of Vif-defective and Vpr-defective viruses was completely restricted. The expression of APOBEC3G (A3G) cytidine deaminase in NKR cells explains why Vif, but not Vpr, was required for HIV-1 replication. When the Vpr-defective virus life cycle was compared with the WT virus life cycle in the semi-permissive cells, it was found that the Vpr-defective virus could enter the cell and produce virions containing properly processed Gag and Env proteins, but these virions showed much less efficiency for reverse transcription during the next-round of infection. In addition, although viral replication was restricted in the non-permissive cells, treatment with arsenic trioxide (As_2_O_3_) could completely restore WT, but not Vpr-defective virus replication. Moreover, disruption of Vpr binding to its cofactor DCAF1 and/or induction of G2 arrest activity did not disrupt the Vpr activity in enhancing HIV-1 replication in NKR cells.

**Conclusions:**

These results demonstrate that HIV-1 replication in NKR cells is Vpr-dependent. Vpr promotes HIV-1 replication from the 2nd cycle likely by overcoming a block at early stage of viral replication; and this activity does not require DCAF1 and G2 arrest. Further studies of this mechanism should provide new understanding of Vpr function in the HIV-1 life cycle.

## Background

The *vpr* gene is highly conserved in the primate lentiviruses, which include HIV-1, HIV-2, and SIV (reviewed in [1]). HIV-2 and some SIV strains additionally express *vpx*, a *vpr* paralog acquired by gene duplication or non-homologous recombination with an ancestral *vpr* gene [2,3]. Both Vpr and Vpx proteins are incorporated into nascent virions at a high copy number via an interaction with Gag and consequently are present in the cytoplasm of the target cells [4-8], indicating that they play a role in the early stage of viral infection. In fact, inactivated *vpr* genes quickly revert back to the active form after infecting a human subject, chimpanzees, and rhesus monkeys, indicating that *vpr* is under strong positive selection [9,10]; *vpr* mutations are frequently found in HIV-1 patients with slow disease progression [11-14]; *vpr*/*vpx* double-deletion mutation markedly attenuates SIV replication in rhesus monkeys [15,16]; *vpx* single-deletion mutation significantly attenuates SIV replication in pig-tailed monkeys [17,18]. These results suggest that *vpr* and *vpx* are very important for viral replication and disease progression in vivo.

HIV-1 Vpr exhibits two major activities in vitro: induction of G2 arrest and enhancement of viral replication in monocyte-derived macrophages (MDMs) (reviewed in [19,20]). Vpx does not induce G2 arrest, but it enhances viral replication in both MDMs and monocyte-derived dendritic cells (MDDCs) [21]. More importantly, Vpx enhances HIV-1 replication *in trans* in these myeloid cells [22,23]. The mechanism of Vpr-induced G2 arrest has been thoroughly studied. Vpr hijacks a host DNA-damage-response (DDR) pathway to trigger G2 arrest by activating the DNA damage sensor ATR but not ATM [24]. In particular, Vpr binds to the DDB1-Cul4A-associated-factor-1 (DCAF1) protein, which is recognized by the Cullin (Cul) 4A E3 ligase consisting of Cul4A, RING H2 finger protein homolog (RBX1), and DNA damage-binding protein 1 (DDB1) (reviewed in [25]). It is currently considered that Vpr triggers proteasomal degradation of an as-yet-unknown cell cycle regulator, resulting in ATR-activation and G2 arrest [25]. The ATR-activation by Vpr also triggers apoptosis [24] and the up-regulation of cell surface protein ULBP2 [26], which is a ligand for the natural killer (NK) cell activation receptor NKG2D. Together, all these downstream events may induce killing of infected cells and contribute to viral pathogenesis in vivo.

Although both Vpr and Vpx enhance HIV-1 replication in MDMs, their levels of enhancement are different, and different mechanisms are involved. While initial experiments showed that Vpr could only enhance HIV-1 replication by 2- to 5 -fold (reviewed in [27]), the activity of Vpx could enhance replication by about 100-fold [28-30]. Vpr has several other activities in cell culture, including activation of HIV-1 long-terminal-repeat (LTR), increase of viral reverse transcription fidelity, and promotion of viral DNA nuclear import [31]. Although all these activities could benefit viral replication, Vpr-enhanced nuclear import seems to be more relevant for the viral replication enhancement [27]. Vpx also enhances viral nuclear import, but it promotes viral replication through DCAF1 by overcoming a restriction factor that blocks viral reverse transcription [29,30]. Recently, SAMHD1 was identified as a myeloid cell-specific HIV restriction factor, which is counteracted by Vpx [32,33].

It has been generally considered that Vpr and Vpx do not promote viral replication in primary or immortalized CD4^+^ T-cells. Nonetheless, several groups have reported some levels of viral promotion: Vpr increases HIV-1 replication in human peripheral mononuclear cells (PBMCs) or purified primary CD4^+^ T-cells by 2- to 6-fold [34-36]; Vpr from a SIV strain that does not encode Vpx enhances SIV replication in PBMCs by more than 10-fold [37]; Vpr and Vpx jointly enhance HIV-2 or SIV replication in the human 174xCEM cell line or a simian T-cell line by 10-fold [38,39]; Vpx alone enhances HIV-2 or SIV replication in PBMCs by more than 10-fold [40,41]. These results strongly argue that Vpr should play a positive role in HIV-1 infection of CD4^+^ T-cells during natural infection. Because CD4^+^ T cells are the primary targets for HIV-1 replication and the loss of these lymphocytic cells is responsible for immunodeficiency, we investigated how Vpr affects HIV-1 replication in these cells. Our efforts result in the identification of human CD4^+^ T-cells where HIV-1 replication is completely dependent on Vpr. These results suggest an important Vpr function in HIV-1 replication, which was not appreciated before.

## Results

### Vpr is required for HIV-1 replication in the permissive and semi-permissive NKR clones

**Figure 1:**
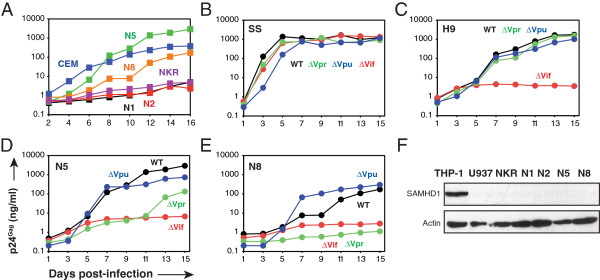
**Vpr is required for HIV-1 replication in the permissive and semi-permissive NKR clones.** Wild-type (WT) HIV-1 (NL4-3) replication was measured in NKR and its subclones by p24^Gag^ ELISA **(A)**. In addition, WT, ΔVif, ΔVpr, and ΔVpu HIV-1 replication was compared in SS **(B)**, H9 **(C)**, N5-P **(D)**, and N8-SP **(E)** cells. Data represent one of at least three independent experiments. **(F)** Endogenous SAMHD1 expression in the indicated cell lines was determined by Western blotting, and the amounts of actin were used as loading control.

Previously, we reported a potent HIV-1 restriction in the human CD4^+^ CEM.NKR (NKR) T cells [42]. NKR cells express both CD4 and CXCR4, but their viral production is typically 100-fold to 1000-fold lower than other human T cells. However, we also found that although the original NKR cells were clonally derived, they contained heterogeneous populations that exhibit different levels of HIV-1 resistance due to unknown variability. From the original NKR cells, we isolated eight NKR subclones that showed three levels of HIV-1 resistance: four clones (N1, N2, N3, N6) were completely non-permissive (NP); two (N7, N8) were semi-permissive (SP); two (N4, N5) were highly permissive (P) [43]. As can be seen, the viral production from N1-NP, N2-NP and the original NKR cells was ~1,000-fold lower than production from N5-P, and ~100-fold lower than production from N8-SP and the original CEM cells (Figure 1A). All these cells grew similarly (data not shown), indicating that the differences in viral production should not result from the differences in cell division.

Because HIV-1 could replicate in N5-P and N8-SP cells, we infected them with WT, Vif-defective (ΔVif), Vpr-defective (ΔVpr), or Vpu-defective (ΔVpu) HIV-1; and as controls, the human CD4^+^ T-cell line H9 and another CEM-derived cell line CEM-SS (SS) were also infected. As expected, it was found that in SS cells, all four viruses replicated equally well (Figure 1B); in H9 cells, only the ΔVif virus did not replicate due to A3G expression [44] (Figure 1C). In N5-P and N8-SP cells, both the WT and ΔVpu viruses replicated well; the ΔVif virus failed completely to replicate in N5-P and N8-SP cells (Figure 1D, Figure 1E). It was not surprising that the ΔVif virus did not grow, because these cells also expressed A3G [43]. However, it was very surprising that the ΔVpr virus replicated very slowly in the N5-P cells, and like the ΔVif virus, it failed completely to replicate in the N8-SP cells (Figure 1D, Figure 1E). Because SAMHD1 was recently identified as a Vpx-sensitive restriction factor and because its expression was not limited to the myeloid tissues [32,33], we wondered whether SAMHD1 played a role in these cells. However, we could not detect SAMHD1 expression in NKR cells and the other clones, but it was detected in THP1 cells (Figure 1F). These results demonstrated that Vpr was required for HIV-1 replication in the permissive and semi-permissive NKR clones and that it did not target SAMHD1.

### Vpr is required for the 2nd round of infection

**Figure 2:**
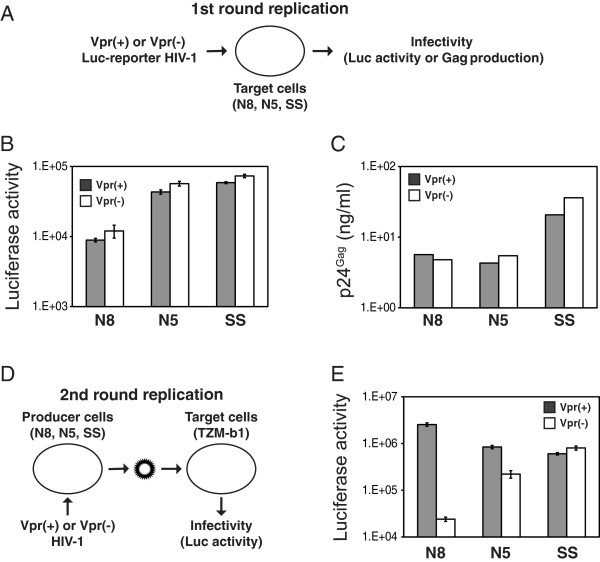
**Vpr is required for the 2nd round of infection. (A)** Schematic illustration of the 1st round viral replication assay. WT or ΔVpr HIV-1 luciferase (Luc)-reporter viruses were produced by transfection of 293T cells with pNL-ΔGag plus pNL-Luc-ΔEnv, or pNL-ΔGagΔVpr plus pNL-Luc-ΔEnvΔVpr vectors. After normalization by p24^Gag^ ELISA, equal amounts of viruses were used to infect indicated cells. Two days later, viral infectivity was determined by measuring the intracellular luciferase activity **(B)**, or viral production from the supernatant by p24^Gag^ ELISA **(C)**. **(D)** Schematic illustration of the 2nd round viral replication assay. Indicated cells were infected with VSV-G pseudotyped WT or ΔVpr HIV-1 (NL4-3) by spinoculation. Three days later, virions were collected from supernatants, and equal amounts of virions were used to infect the HIV indicator TZM-b1 cells. Thirty-six hours later, viral infectivity was determined by measuring the intracellular luciferase activity **(E)**. The standard errors (S.E.) in (B) and (E) were calculated from three independent experiments.

We then determined how Vpr promoted viral replication in N8-SP and N5-P cells. First, we determined whether Vpr was required for the 1st round of infection. HIV-1 luciferase (Luc) reporter viruses that only replicated one cycle were produced from 293T cells in the presence or absence of Vpr; their infectivity was measured in N8-SP, N5-P, and SS cells (Figure 2A). It was found that both Vpr(+) and Vpr(−) viruses produced similar levels of luciferase activity in these cells (Figure 2B), and viral production from these cells in the presence or absence of Vpr was also quite similar (Figure 2C). These results suggested that Vpr was not required during the 1st round of viral replication.

Second, we determined whether Vpr was required for the 2nd round of infection. N8-SP, N5-P, and SS cells were infected with WT or ΔVpr virus. Newly produced viruses were collected, and their infectivity was measured by infection of the HIV indicator TZM-b1 cells (Figure 2D). It was found that Vpr did not increase HIV-1 infectivity in SS cells, but it increased infectivity significantly in N8-SP cells and less significantly in N5-P cells (Figure 2E). These results suggested that Vpr is required for the 2nd round of viral replication.

### Vpr enhances an early stage of viral replication at the 2nd round of infection

**Figure 3:**
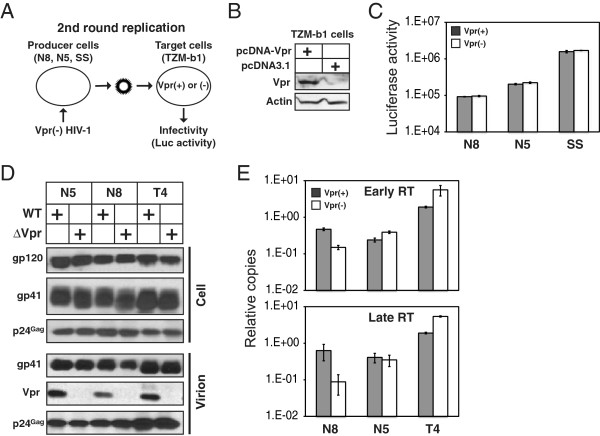
**Vpr enhances an early stage of viral replication at the 2nd round of infection. (A)** Schematic illustration of the rescue of viral replication assay by Vpr in target cells. Indicated cells were infected with VSV-G pseudotyped ΔVpr HIV-1 (NL4-3) by spinoculation. Three days later, virions were collected from supernatants, and equal amounts of virions were used to infect the HIV indicator TZM-b1 cells, which were transfected with a Vpr expression or control vector. The Vpr expression in TZM-b1 cells were determined by Western blotting **(B)**, and viral replication in TZM-b1 cells was determined by measuring the intracellular luciferase activity **(C)**. **(D)** To analyze viral protein expression, indicated cells were infected VSV-G pseudotyped WT or ΔVpr HIV-1 by spinoculation. Two days later, virions were purified from the culture supernatants by ultracentrifugation. Viral protein expression in infected cells and virions was detected by Western blotting. **(E)** To analyze viral early and late reverse transcription (RT), T4 cells were infected with WT or ΔVpr HIV-1 purified from spinoculated N5-P, N8-SP, and T4 cells, and 12 hours later, viral early and late RT products were quantitated by real-time PCR. Copy numbers were presented as relative values after normalization with mitochondrial DNA. The standard errors (S.E.) in (C) and (E) were calculated from three independent experiments.

We next investigated how Vpr enhanced viral replication during the 2nd round of infection. Since Vpr was expressed in the producer cells in the previous experiment, we expressed Vpr in the target cells, and tested whether it could rescue the ΔVpr virus replication. TZM-b1 cells were transfected with a pcDNA3.1 vector expressing codon-optimized Vpr gene or an empty vector, and these cells were infected with ΔVpr HIV-1 produced from N8-SP, N5-P, and SS cells (Figure 3A). The expression of Vpr in TZM-b1 cells was clearly detected (Figure 3B). However, even in the presence of Vpr, the ΔVpr HIV-1 infectivity did not increase in the target cells (Figure 3C). This result suggested that Vpr should be expressed from the producer cells to rescue the 2nd round of HIV-1 replication in NKR cells.

Next, we determined whether the N8-SP and N5-P cells produced defective particles in the absence of Vpr, which would reduce viral infectivity during the 2nd cycle of viral replication. N8-SP, N5-P, and another CEM-derived CEM-T4 (T4) cells were infected with WT or ΔVpr virus; virions were purified from culture supernatants by ultracentrifugation; Gag, Env, and Vpr expression in cells and virions was determined by Western blotting. We confirmed that HIV-1 replication in T4 cells did not require Vpr (data not shown). It was found that similar levels of processed Gag (p24) and Env (gp120, gp41) were detected in both infected cells and virions regardless of Vpr expression (Figure 3D). These results suggested that these cells did not produce structurally defective virions in the absence of Vpr, and neither did Vpr affect Gag, Pol, and Env expression.

Lastly, we analyzed the early stage of viral replication during the 2nd cycle of infection. T4 cells were infected with WT or ΔVpr virus purified from HIV-1-infected N5-P, N8-SP, or T4 cells; twelve hours later, viral early and late reverse transcription (RT) products were chased by real-time PCR. It was found that the ΔVpr virus from T4 cells generated slightly more early and late viral RT products than the WT virus (Figure 3E). In contrast, the ΔVpr virus from N5-P cells generated similar levels of both RT products as the WT virus, whereas the ΔVpr virus from N8-SP cells generated 3- to 8-fold less of both RT products than the WT virus. These results suggested that Vpr should enhance an early stage of viral replication during the 2nd cycle infection of these cells.

### Vpr is also required for HIV-1 replication in the non-permissive NKR clones

**Figure 4:**
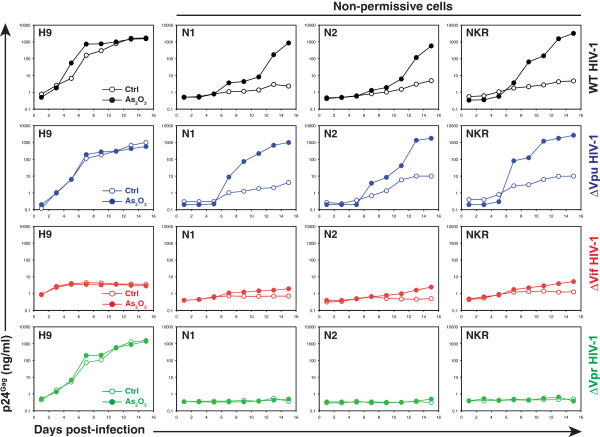
**Vpr is required for HIV-1 replication in the parental NKR cells and non-permissive clones.** H9, NKR, N1-NP, and N2-NP cells were infected with WT, ΔVif, ΔVpr, or ΔVpu HIV-1 (NL4-3) virus in the presence or absence of 0.2 μM As_2_O_3_. Viral replication in these cells was detected by p24^Gag^ ELISA. All experiments were repeated at least twice and consistent results were obtained.

Furthermore, we determined whether Vpr was required in the non-permissive clones as well as the parental NKR cells. Because these cells were highly refractory to HIV-1 infection, we first established a method to restore viral replication. It was reported that arsenic could increase HIV-1 replication in human cells, although the mechanism for this activity remains unclear [45,46]. We tested arsenic activity in these NKR cells by treatment with As_2_O_3_. As_2_O_3_ is very toxic to human T cells because of its ability to induce apoptosis [47,48]; so a very low concentration (0.2 μM) was used. Surprisingly, a completely recovery of WT HIV-1 replication was found in NKR, N1-NP, and N2-NP cells after this treatment (Figure 4, top panels). In contrast, the same treatment did not apparently affect WT HIV-1 replication in the H9 cells. We then compared ΔVif, ΔVpr, and ΔVpu virus replication in these treated cells. In H9 cells, the ΔVif virus was the only one that did not grow, and the As_2_O_3_ treatment had little influence on the replication of these three viruses (Figure 4). In NKR, N1-NP, and N2-NP cells, only the ΔVpu virus grew well in the presence of the As_2_O_3_ treatment; even under such treatment, both ΔVif and ΔVpr viruses did not grow (Figure 4). Thus, Vpr was also required for HIV-1 replication in the parental NKR cells and the non-permissive clones.

**Figure 5:**
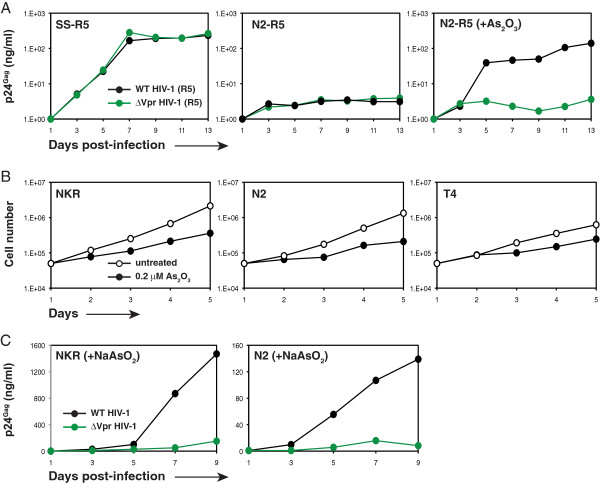
**Viral tropism and Vpr-dependency.** (**A**) SS-R5, N2-R5, and arsenic-treated N2-R5 cells were infected with R5-tropic WT or ΔVpr HIV-1 (NLAD8), and viral replication was detected by p24^Gag^ ELISA. Data represent one of at least three independent experiments. **(B)** Measurement of cell growth rate under As_2_O_3_ treatment. Indicated cells were treated with 0.2 μM As_2_O_3_ or untreated. Cell numbers were counted daily after staining with trypan blue. **(C)** Effect of NaAsO_2_ on HIV-1 replication. Indicated cells were infected with WT or ΔVpr HIV-1 in the presence of 2 μM NaAsO_2_ and viral replication was detected by p24^Gag^ ELISA.

To understand whether the Vpr-dependent HIV-1 replication was affected by viral tropism, we created a N2-NP cell line expressing human CCR5 (N2-R5). A similar cell line from SS was also created (SS-R5) to be the control. These cells were infected with R5-tropic WT or ΔVpr HIV-1 strain NL-AD8, and viral replication was determined. It was found that both WT and ΔVpr NL-AD8 viruses replicated well in the SS-R5 cells, but they failed completely to grow in the N2-R5 cells (Figure 5A). When the N2-R5 cells were treated with As_2_O_3_, the WT virus started to replicate, but the ΔVpr virus did not (Figure 5A). Thus, the Vpr-dependency was not influenced by viral tropism.

To understand whether the enhancement of HIV-1 replication by As_2_O_3_ was due to speeding up cell division, we compared the growth of NKR, N2-NP, and T4 cells in the presence or absence of 0.2 μM As_2_O_3_ treatment. It was found that As_2_O_3_ slightly reduced their growth rate, indicating that it might have some toxic effect at this concentration (Figure 5B). We also tried another less-toxic arsenic compound (NaAsO_2_). NKR and N2-NP cells were infected with WT or ΔVpr HIV-1 in the presence of 2 μM NaAsO_2_ and viral replication was measured. It was found that like the As_2_O_3_ treatment, the NaAsO_2_ treatment also selectively increased the WT, but not the ΔVpr HIV-1 replication (Figure 5C). These results suggested that the effect of arsenic in these cells was specific.

### DCAF1 and G2 arrest are not required for Vpr enhancement of viral replication

As introduced earlier, Vpr interacts with DCAF1 to induce G2 arrest; although Vpx does not cause G2 arrest, it interacts with DCAF1 to neutralize SAMHD1. We wondered whether Vpr enhancement of viral replication required DCAF1 and/or G2 arrest. We introduced two well-characterized mutations (Q65R, R80A) into the *vpr* gene in the proviral clone pNL4-3. The Vpr Q65R mutant does not bind to DCAF1 and therefore does not induce G2 arrest [49]; although the R80A mutant binds to DCAF1, it does not induce G2 arrest [26,50].

**Figure 6:**
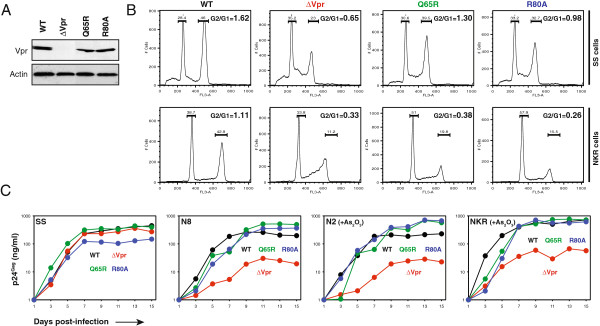
**Vpr mutagenesis analyses. (A)** Analysis of Vpr protein expression in viral producer cells. HIV-1 proviral vectors expressing indicated Vpr proteins were transfected into 293T cells, and Vpr expression was determined by Western blotting. The amounts of actin were used as loading control. **(B)** Analysis of cell cycle. SS and NKR cells were infected with HIV-1 expressing indicated Vpr proteins. After two days, intracellular Gag proteins were stained with fluorescent antibodies and intracellular DNAs were stained with propidium iodide. The Gag-positive population was sorted and analyzed for cell cycle profiles by flow cytometry. The G2/G1 ratios were calculated by dividing the proportion of cells in G2 and M phases with that in G0 and G1 phases. **(C)** Replication of HIV-1 bearing Vpr mutations. SS, H9, N8-SP, and As_2_O_3_-treated N2-NP [N2 (+As)] and NKR [NKR (+As)] cells were infected with HIV-1 expressing indicated Vpr proteins. Viral replication was detected by p24^Gag^ ELISA. All experiments were repeated at least twice and consistent results were obtained.

First, we compared the expression and activity of these Vpr proteins. The WT, ΔVpr, Q65R, and R80A viruses were produced by transfecting 293T cells with these proviral constructs. The Vpr expression in 293T cells was determined by Western blotting; the G2 arrest activity was determined by infection of SS and NKR with these viruses. It was found that both Vpr Q65R and R80A mutants were expressed at similar levels as the WT protein (Figure 6A). In addition, the G2/G1 ratio in ΔVpr and WT virus-infected cells shifted from 0.33 to 1.11 in the parental NKR cells and from 0.65 to 1.62 in SS cells, indicating that Vpr induced strong G2 arrest in these cells (Figure 6B). The background levels of G2 arrest in the ΔVpr virus-infected cells were likely caused by Vif, which also has similar activity [51]. Compared to the WT virus, the levels of G2 arrest by the Q65R mutant in SS and NKR cells were significantly reduced, and these levels were further reduced in the R80A mutant virus-infected cells (Figure 6B). These results were consistent with previous observations made by other investigators [26,49,50].

Second, we measured viral replication in SS, N8-SP, N2-NP, and the parental NKR cells. Because the N2-NP and parental NKR cells were highly non-permissive, these cells were treated with As_2_O_3_ during infection. It was found that in the SS cells, all these viruses replicated equally well; in the N8-SP and arsenic-treated N2-NP and NKR cells, only the ΔVpr virus replicated poorly, whereas the WT, Q65R, and R80A viruses replicated almost equally well (Figure 6C). These results suggested that both G2 arrest and DCAF1-binding should not be required for Vpr enhancement of viral replication in NKR cells.

## Discussion

In this report, we present compelling evidence to demonstrate that Vpr strongly enhances HIV-1 replication in the human CD4^+^ NKR T-cells. The ΔVpr virus only produces baseline levels of virions in the semi-permissive clone N8-NP, non-permissive clones N1-NP and N2-NP, and parental NKR cells (~1 to 10 ng/ml p24^Gag^), whereas the WT virus produces very high levels (100–1000 ng/ml p24^Gag^) (Figure 1 and Figure 4). These results suggest that Vpr could enhance HIV-1 replication by 100- to 1000-fold in these cells, which is a much greater effect than the previously reported Vpr effect in macrophages and other CD4^+^ T-cells. In fact, this Vpr effect is at the same level as that seen with Vif, highlighting its important role in viral infection of CD4^+^ T-cells.

The mechanism of Vpr enhanced viral replication reported here is different from what was reported before. As introduced earlier, Vpr was shown to facilitate nuclear import of viral DNA in macrophages, which was considered as a major mechanism for HIV-1 replication enhancement [27]. We found that Vpr was not required for viral replication during the 1st cycle of viral replication in NKR cells (Figure 2B, Figure 2C), indicating that it did not promote viral replication at this step. Indeed, Vpr was required for the 2nd cycle of viral replication (Figure 2E). Vpr did not affect Gag and Env expression and processing, and it also had no effect on Env packaging (Figure 3D), indicating that it should not play a role in viral entry. Nevertheless, the ΔVpr virus exhibited poor efficiency in conducting reverse transcription, indicating that viral replication should be blocked at an early stage after entering into the cell. Using the parental NKR cells, our previous work suggested that NKR cells should express a dominant factor that inhibited the WT virus replication from the 2nd cycle [42]. Since arsenic could completely restore the WT viral replication, this inhibitor was likely disrupted by arsenic. This arsenic-sensitive inhibitor should not exist in the permissive and semi-permissive clones, because the WT HIV-1 replicated well in these cells without arsenic treatment. Nevertheless, all NKR cells, with a possible exception for the permissive clone N5-P, should express another Vpr-sensitive inhibitor at high levels, because viral replication in these cells was Vpr-dependent. Our results suggest that this unknown factor should be packaged into virions and block an early stage of viral replication at the 2nd round of infection, because we found that Vpr was required to be expressed in the viral producer cells and rescued viral reverse transcription in the target cells (Figure 2E, Figure 3C, Figure 3E). Recently, a genome-wide siRNA screening identified 52 new host factors that could inhibit HIV-1 replication at the early stages of viral life cycle [52]. It will be interesting to know how they are expressed in NKR cells and whether they are targeted by Vpr. Alternatively, Vpr may also recruit a positive cellular factor from the viral producer cells into HIV-1 virions that promotes the early stage of viral replication, although we showed before that NKR cells should not be deficient in host factors to support HIV-1 replication [42].

To understand how Vpr counteracted this factor, we tried to disrupt Vpr and DCAF1 interaction by introducing the Q65R mutation, and we found that this mutant was still able to enhance viral replication (Figure 6C). We also made the R80A mutant that still binds DCAF1 but does not induce G2 arrest according to the literatures, and we found that it was also capable of enhancing viral replication (Figure 6C). These results strongly suggest that DCAF1 and G2 arrest are not required for this Vpr activity. As introduced earlier, Vpx neutralizes the restriction factor SAMHD1, and this activity is DCAF1-dependent [32]. However, there is another unknown HIV-1 restriction factor in MDDCs, which is neutralized by Vpx in a DCAF1-indepednent manner [53]. In addition, it has been reported that Vpr could enhance HIV-1 replication in the human Hut78 T-cell line and this activity was independent of DCAF1 [36]. Thus, the Cul4A E3 ligase is not always required for Vpr and Vpx activity.

## Conclusion

We have identified human CD4^+^ T-cells where HIV-1 replication is completely dependent on Vpr. Vpr promotes HIV-1 replication in NKR cells from the 2nd round of infection, likely by overcoming an early block; and its activity does not require DCAF1 and G2 arrest. We suggest that further study of the Vpr activity in NKR cells will provide new understanding of Vpr function in the HIV-1 life cycle and uncover a novel anti-retroviral mechanism.

## Methods

### Cells

The HIV reporter cell line TZM-b1, human T-cell lines H9, Jurkat, CEM.NKR, and CEM-SS, and human monocytic cell line THP1 were obtained from NIH AIDS Research and Reference Reagent Program. 293T, U937, and the original CEM cells (CCRF-CEM) were purchased from ATCC. The CEM.NKR subclones N1, N2, N5, and N8 were described before [43]. All T-cell and monocytic cell lines were cultured in RPMI 1640 with 10% fetal bovine serum (HyClone). 293T, and TZM-bI cells were cultured in DMEM with 10% bovine calf serum (HyClone).

To stably express human CCR5 in N2 and CEM-SS cell lines, recombinant retrovirus expressing human CCR5 was produced by transfection of 293T with retroviral vector pBABE.CCR5, packaging vector pCgp, and VSV-G expression vector. Cells were then infected with the virus and stable cell lines were selected by puromycin (0.5 μg/ml).

### Plasmids

The HIV-1 proviral constructs pNL-ΔVif, pNL-ΔVpr*,* and pNL-ΔVpu were obtained from K. Tokunaga; pNL-AD8 was obtained from E. Freed; pCgp was obtained from P. Cannon; pBABE.CCR5 was obtained from N. Landau through the NIH AIDS Research and Reference Reagent Program; pcDNA3.1 expressing codon-optimized Vpr was obtained from M.J. Lenardo [54]. The wild-type pNL4-3, the HIV-1 Env expression vector pNL-ΔGag, and luciferase-reporter vector pNL-LucΔEnv were described before [42,55]. pNL-AD8-ΔVpr, pNL-ΔGagΔVpr, and pNL-Luc-ΔEnvΔVpr vectors were created by swapping the AgeI/EcoRI fragment with the pNL-ΔVpr vector. The Vpr Q65R and R80A mutations were introduced into pNL4-3 by site-directed mutagenesis.

### Virus production

HIV-1 virions were produced from 293T cells by the standard calcium phosphate transfection. Typically, 20 μg proviral DNA were used to transfect 293T cells cultured in a 100-mm dish with 40% confluence, and viruses were collected from the supernatants after 48 hours. Viral production was measured by p24^Gag^ ELISA.

### HIV-1 infection of human T cell lines

A total of 2 × 10^5^ cells were incubated with equal amounts of virus at 37°C for three hours. After removal of the inocula and washing three times, cells were cultured in 24-well plates for 16 days. Culture supernatants were then collected and replaced with new medium every other day, and viral production was measured by p24^Gag^ ELISA. For spinoculation, cells were placed in a 48-well plate with the virus and centrifuged at 1,200 × g, 25°C, for 2 hours. Cells were washed and viral production was determined similarly. After two days, viruses were harvested from supernatants and purified by ultracentrifugation at 222,000 x g, 4°C, for 30 min. Virions were then collected for Western blot analysis.

### Real-time PCR analysis of viral cDNAs

A total amount of 200 ng virions purified from N5-P, N8-SP, and T4 cells after spinoculation were inoculated into 2 × 10^6^ T4 cells at 37°C for 2 hours. Cells were then washed with phosphate-buffered saline (PBS) and cultured for additional 12 hours. The total cellular DNAs were extracted from these cells by the DNeasy kit (Qiagen), and purified DNAs were further treated with DpnI at 37°C for 1 hour to remove any plasmid DNA contamination. Equal amounts of cellular DNAs were used for real-time PCR using TaqMan® Universal PCR Master Mix kit (Applied Biosystems). The early reverse transcripts (strong stop) were amplified by primers oHC64/oHC65 and quantitated by a fluorescence labeled probe oHC66; the late reverse transcripts were amplified by MH531/MH532 and quantitated by LRT-P; mitochondrial DNA were amplified by MH533/MH 534 and quantitated by mito-probe [56]. Reactions were performed in triplicate. After initial incubation at 95°C for 10 minutes, 40 cycles of amplification were carried out for 15 sec at 95°C followed by 1 minute at 60°C. Reactions were analyzed using a 7900HT system (Applied Biosystems). Finally, relative HIV-1 cDNA copies were calculated by normalization to the levels of mitochondrial DNA.

### Measurement of intracellular luciferase activity

After a 36-hour infection, cells were lysed in a buffer (25 mM Tris–HCl, pH 7.8, 2 mM dithiothreitol, 2 mM 1,2-diaminocyclohexane-N,N,N',N'-tetraacetic acid, 10% glycerol, 1% Triton X-100). After removing the nuclei, the cytosolic fraction was used to determine the firefly luciferase activity with a luciferase assay kit (Promega).

### Cell cycle analysis

1 × 10^6^ cells were infected with HIV-1 with or without Vpr mutations, respectively. Two days later, cells were harvested and washed once with cold PBS. Washed cells were resuspended in 1 ml of cold PBS, and then slowly added into 9 ml of ice-cold 70% ethanol with gently vortexing. Ethanol-fixed cells were left overnight at −20°C. The following day, cells were centrifuged at 500 × *g* to remove ethanol and cells were washed with cold PBS containing 0.1% Titon X-100 (PBS-T). Cells were then incubated with 30 μl cold PBS-T containing 1 μl of anti-Gag antibody (183-H12-5C) for 30 minutes. After washing two times with PBS-T, cells were incubated with 30 μl cold PBS-T containing 1 μl of FITC-conjugated anti-mouse immunoglobulin antibody for another 30 minutes. After further washing, cells were resuspended in PBS-T staining buffer containing 20 μg/ml propidium iodide and 200 μg/ml RNase, and allowed to incubate for two hours on ice. Cell cycle profiles were analyzed by flow cytometry and results were analyzed by FlowJo to derive percentages of cells in different phases of cell cycle.

### Western blotting

The anti-SAMHD1 antibody was perchased from Proteintech Group. HIV-1 viral proteins were detected by antibodies from NIH AIDS Research and Reference Reagent Program and their catalogue numbers are: 1513 (Gag), 526 (gp41), 521 (gp120), and 11836 (Vpr). Horseradish peroxidase (HRP)-conjugated anti-goat, rabbit, or mouse immunoglobulin G secondary antibodies were purchased from Pierce. Detection of the HRP-conjugated antibody was performed using an enhanced chemiluminescence detection kit (Amersham Bioscience).

## Abbreviations

HIV-1 or 2: Human immunodeficiency virus type 1 or 2; Vpr: Viral protein regulatory; NKR: the parental CEM.NKR cells; WT: Wild-type; P: Permissive; SP: Semi-permissive; NP: Non-permissive; A3G: APOBEC3G; SIV: Simian immunodeficiency virus; MDM: Monocyte-derived macrophage; MDDC: Monocyte-derived dendritic cells; DDR: DNA-damage-response; ATM: Ataxia telangiectasia mutated kinase; ATR: ATM- and Rad3-related kinase; Cul4A: Cullin 4A; RBX1: RING H2 finger protein homolog 1; DDB1: DNA damage-binding protein 1; DCAF1: DDB1-Cul4A-assoicated factor 1; NK: Natural killer; Luc: Luciferase; NKG2D: Natural Killer Group 2D; ULBP2: Unique long 16-binding protein 2; siRNA: Small interfering RNA; PBS: Phosphate-buffered saline.

## Competing interests

The authors declare that they have no competing interests.

## Authors’ contributions

TZ, YD, JJB, and JZ conducted the experiments and analyzed the data. TZ and YHZ conceived the study, and YHZ supervised the entire project. YHZ wrote the manuscript. All authors read and approved the final manuscript.
